# Single-stage posterior debridement, decompression and transpedicular screw fixation for the treatment of thoracolumbar junction (T12-L1) tuberculosis with associated neurological deficit: a multicentre retrospective study

**DOI:** 10.1186/s12891-019-2466-7

**Published:** 2019-03-04

**Authors:** Yanping Zeng, Wenjie Wu, Jingtong Lyu, Xun Liu, Jiulin Tan, Zhilin Li, Yuan Chen, Litao Li, Yonghong Zheng, Gaoju Wang, Jianzhong Xu, Zehua Zhang

**Affiliations:** 1 Department of Orthopaedics, Southwest Hospital, Third Military Medical University, Chongqing, 400038 China; 2 Department of Orthopaedics, The Lanzhou General Hospital, Lanzhou Military Command of CPLA, Lanzhou, China; 3 Department of Orthopaedics, Yulin People’s Hospital, Yu Lin, China; 4 Department of Orthopaedics, The People’s Liberation Army No. 309 Hospital, Beijing, China; 5 Department of Orthopaedics, Xi’an Jiaotong University Affiliated Honghui Hospital, Xi’an, China; 6 Department of Orthopaedics, The Affiliated Hospital of Southwest Medical University, Luzhou, China

**Keywords:** Spinal tuberculosis, Thoracolumbar junction, Neurological deficit, Posterior decompression

## Abstract

**Background:**

A multicentre retrospective study was conducted to evaluate the safety and efficacy of single-stage posterior debridement, decompression and transpedicular screw fixation for the treatment of thoracolumbar junction (T12-L1) tuberculosis in patients with associated neurological deficit.

**Methods:**

Thoracolumbar junction (T12-L1) tuberculosis patients (*n* = 69) with neurological deficit who underwent single-stage posterior debridement, decompression and transpedicular screw fixation from January 2005 to January 2015 were included in the study. Antituberculosis therapy was performed both before and after surgery. The surgery duration and patient blood loss were evaluated, in addition to the change in pain visual analogue score (pVAS), kyphotic angle, Oswestry disability index (ODI) score and American Spinal Injury Association (ASIA) grade assessed preoperatively, immediate postoperatively and at the final follow-up visit.

**Results:**

The average blood loss was 354 ± 291 mL. The average kyphosis angle was corrected from 21 ± 9° preoperatively to 9 ± 4° postoperatively, with a mean decrease in pVAS and ODI scores of 3.4 and 16, respectively. The postoperative ASIA grading was grade A for five patients, grade C for 15 and grade D for 49 patients, which had improved to grade C for four patients, grade D for three patients and grade E for 62 patients at the final follow-up. The neurological deficit did not worsen in any of the patients.

**Conclusions:**

Single-stage posterior debridement, decompression and transpedicular screw fixation is an effective treatment method in thoracolumbar junction (T12-L1) tuberculosis patients with neurological deficit, with good neurological recovery and no progression of kyphosis.

## Background

Tuberculosis poses a serious impact to human health, particularly in developing countries [1–3]. China still has the second greatest number of infected people worldwide [4]. The thoracolumbar spine is one of the major targets of metastatic tuberculosis of the musculoskeletal system [5]. It most commonly affects the thoracolumbar junction [6], and about 10–43% of patients with spinal tuberculosis have associated neurological complications [7]. Spinal tuberculosis may result in spinal deformities and paralysis, and it represents one of the most dangerous pathologies affecting the musculoskeletal system [8].

Chemotherapy and surgery can both achieve satisfactory results with timely diagnosis and long-term treatment [4]. Conservative treatment with antituberculous drugs and external immobilisation remain the preferred treatment options in most cases of spinal tuberculosis [9–12]. Mild spinal tuberculosis can be treated with standard chemotherapy alone [13]. Surgery is necessary for cases of spinal tuberculosis with spinal instability, neurological deficit and severe and/or progressive kyphosis deformity, as well as for patients who do not respond to chemotherapy or those with a large paraspinal abscess [14]. Suitable surgical intervention in a timely manner can improve the stability of the spine, eliminate compression on the spinal cord and prevent the progression of deformity, paralysis or death. Various surgical approaches have been used for the treatment of patients with spinal tuberculosis, including an anterior, anterio-posterior or posterior only approach [15–17]. However, the optimum surgical approach remains uncertain amongst surgeons. Traditionally, the anterior spinal approach has been preferred for cases where the vertebral bodies and disc spaces are the main sites affected by tuberculosis as this approach provides direct access to the infection foci, making it convenient for debridement and reconstruction [18, 19]. However, advances in instrumentation systems and techniques have led to the increasing popularity of the posterior approach for the correction of kyphosis and spine stabilisation [20–22]. This approach has been proven to be effective for the treatment of many thoracolumbar and lumbar spinal disorders that lead to segmental instability [23]. In recent years, the treatment strategy for spinal tuberculosis has become more conservative and less invasive [24].

Although the use of the posterior approach for patients with spinal tuberculosis is widespread, to our knowledge, no studies have reported the effectiveness and safety of single-stage posterior debridement, decompression and transpedicular screw fixation for monosegmental spinal tuberculosis in patients with neurological deficit, focusing on the thoracolumbar junction (T12-L1) only. In this study, we report our findings regarding the treatment of patients with thoracolumbar junction tuberculosis and a related neurological deficit by this approach.

## Methods

The records of 33 men and 36 women who underwent single-stage posterior debridement, decompression and transpedicular screw fixation for thoracolumbar junction (T12-L1) tuberculosis with associated neurological deficit between January 2005 and January 2015 from six hospitals across China were reviewed. Patients were followed up for a minimum of 24 months. The average patient age was 35.06 ± 8.86 years (range 16–53 years). In this study, we did not include patients with HIV co-infection.

The diagnosis of spinal tuberculosis was made based on clinical symptoms, physical signs, laboratory findings and radiological evidence. It was confirmed histologically after debridement in surgical patients. The ASIA score was used to evaluate the neurological function of patients, with five patients preoperatively classified as grade A, 15 patients as grade C and 49 patients as grade D. pVAS was used to evaluate several clinical factors including back pain for all patients. The Cobb technique was used to assess the local kyphotic angle. The ODI score was used to evaluate the functional outcome of patients. Only patients who had thoracolumbar junction tuberculosis with a neurological deficit were included in this study. Those patients who received conservative treatment, who were not affected in the T12-L1 segmental vertebrae only, or who could not undergo vertebral fixation were excluded from the study.

### Preoperative management

All cases were administered a chemotherapy regimen (isoniazid 300 mg/day, rifampicin 450 mg/day, ethambutol 750 mg/day and pyrazinamide 750 mg/day) for 2–4 weeks prior to the operation. Preoperative haemoglobin and ESR levels needed to be higher than 100 g/L and no higher than 40 mm/L, respectively, before surgery.

### Operation technique

All patients underwent general endotracheal anaesthesia, after which they were placed in the prone position on the spinal Table. A standard dorsal midline incision was performed, and the posterior tissues were exposed. Pedicle screws were inserted, and the correct placement was confirmed by intraoperative C-arm fluoroscopy. In general, the two superior and two inferior healthy vertebrae were embedded into the pedicle screw to ensure rigid spinal stability. A temporary pre-bent rod was installed on the side where the lesion was relatively mild in order to avoid spinal cord injury during decompression and focal debridement. A unilateral or bilateral Laminectomy was done according to the extent of cord compression. Then facetectomy and pediculectomy were performed at appropriate levels. The surgical area was washed with hydrogen peroxide and a large quantity of saline. Any abscesses, granulation tissue, sequestra, caseous necrosis, necrotic endplates and discs were debrided as thoroughly as possible via the transpedicular space. Then, a pre-bent rod was temporarily installed on the other side and the rod installed previously was removed. The same process was performed on the side opposite to the lesion. Posterolateral fusion using autograft of suitable size or a titanium cage containing cancellous bone from the iliac crest was performed. The compression and expansion of the internal fixation instrument was used to rectify the kyphosis and scoliosis gradually and carefully, then the contoured rods were tightened. Finally, streptomycin (1.0 g) and isoniazid (0.3 g) were locally administered, and a local drainage tube was placed in the operation site before the incision was closed.

### Postoperative care

The drainage tube was removed when the amount of postoperative drainage was less than 10 ml/day. Preventive antibiotic treatment was administered during the first 48 h after the operation. All patients were recommended to wear the bracing apparatus until bony fusion was observed by radiography. Patients resumed oral Isoniazid, rifampicin, ethambutol, and pyrazinamide (HREZ) chemotherapy postoperatively, then pyrazinamide was discontinued at 6 months. Patients continued to receive a regimen of HRE chemotherapy for 9 to 12 months (6HREZ/9-12HRE) [25]. The liver function and ESR rates of the patients were carefully monitored at regular intervals. Follow-up examinations were conducted at 1, 3, 6, 12 and 18 months postoperatively. Subsequent follow-up examinations were performed at 12-month intervals.

### Statistical analysis

Continuous data are expressed as $\overline{\mathrm{X}}$ ± standard deviation (SD). One-way ANOVA was used to evaluate the difference between preoperatively and postoperatively in ESR, pVAS score, ODI and kyphotic angle. Statistical analyses were performed using SPSS version 22 (SPSS, Inc., Chicago, USA). For all analyses, *P*-values less than 0.05 were considered significant.

## Results

Patients were aged between 16 and 53 years old, with an average age of 35.06 ± 8.86 years at the time of surgery. The mean average blood loss volume was 354 ± 291 mL (range 200–2200 mL). The average follow-up period was 29.17 ± 6.33 months (range 24–52 months) (Table 1), with no patients lost to follow-up and no deaths. There were no cases of non-union of bone, pseudarthrosis, internal fixation loosing or relapse at the final follow-up.

**Table 1:** General data of study

Male/Femal	Age(years)	Follow-up time (mon)	Blood loss (ml)	Hospitalization day (days)
33/36	35.06 ± 8.86	29.17 ± 6.33	354 ± 291	26.36 ± 7.04

The ESR values of patients measured preoperatively, postoperatively and at the last follow-up are presented in Table 2. The average preoperative ESR value was 36 ± 22 mm/h (range 2–99 mm/h), and the average values measured postoperatively and at the final follow-up were 6 ± 5 mm/h (range 0–20 mm/h) and 6 ± 3 mm/h (range 0–15 mm/h), respectively, which were within the normal range. Statistical analysis showed that there was a significant difference between the preoperative ESR values and those recorded 12 weeks postoperative (*P* < 0.05). Significant differences in ESR also existed between 12 weeks postoperative and the final follow-up (*P* < 0.05).

**Table 2:** Clinical details of surgery

Schedule	ESR (mm/h)	VAS	ODI	Kyphosis angle (°)
Preoperative	36 ± 22	6.0 ± 1.5	36 ± 9	21 ± 9
postoperative	6 ± 5	2.6 ± 0.9	20 ± 8	9 ± 4
Final follow up	6 ± 3	0.5 ± 0.6	4 ± 3	10 ± 4

*VAS* visual analogue scale of pain, *ESR* erythrocyte sedimentation rate, Oswestry disability index (ODI) score

**Fig. 1:**
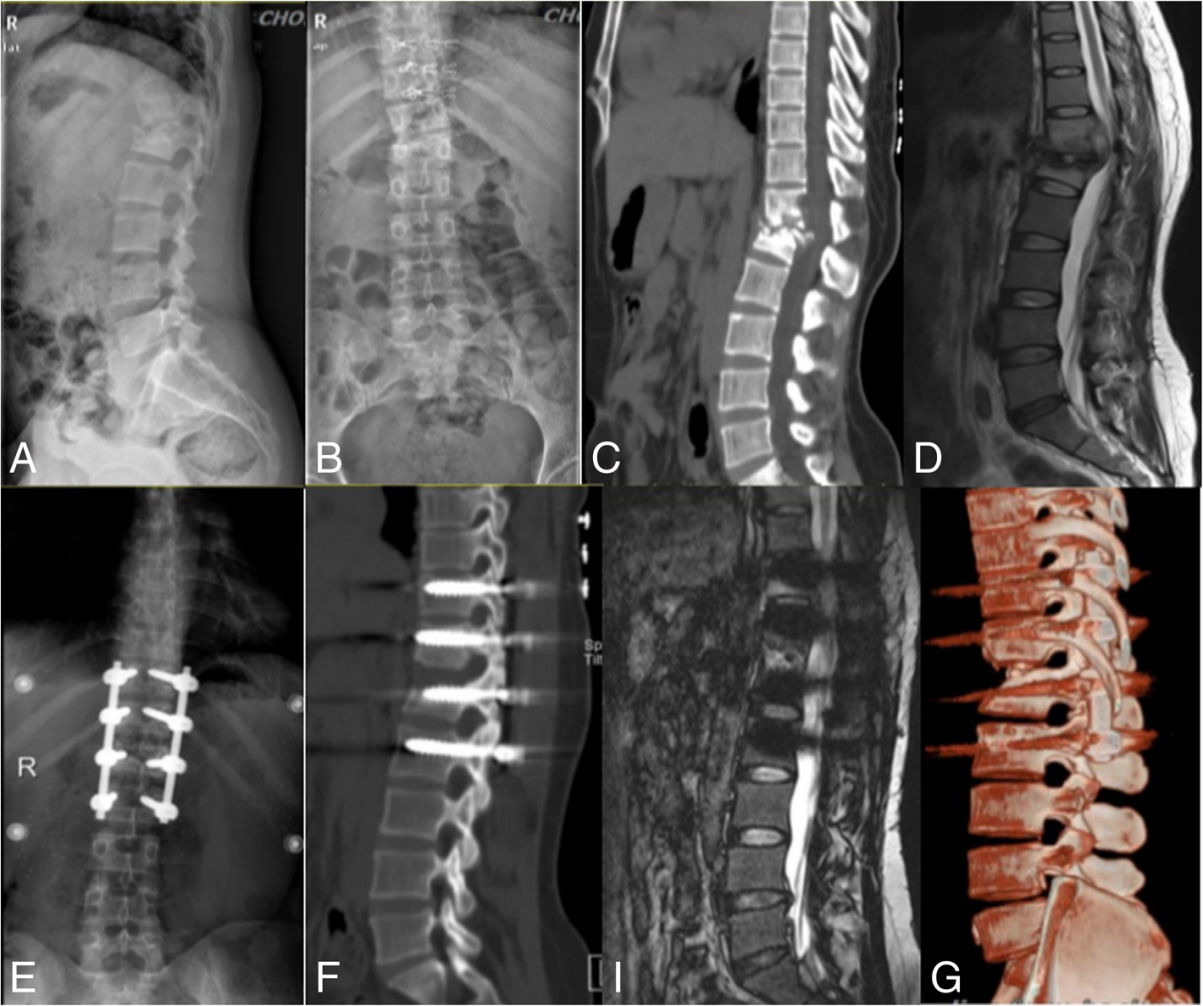
Single-stage posterior debridement, decompression and transpedicular screw fixation of a 35-year-old woman with thoracolumbar junction tuberculosis with associated neurological deficit. (**a**, **b**) Preoperative lateral and anteroposterior X-rays; (**c**) preoperative computed tomography (CT); (**d**) preoperative MRI; (**e**) X-ray at 3 days postoperative; (**f**) CT scan at 12 months postoperative; (**i**) MRI at 12 months postoperative; and (**g**) 3D reconstruction of CT at 12 months postoperative.

The average preoperative and postoperative spinal kyphotic Cobb’s angles were 21 ± 9° and 9 ± 4°, respectively, and this difference was significant (*P* < 0.05). The kyphosis angle was maintained at 10 ± 4° on average at the final follow-up visit. Statistical analysis demonstrated that there was a significant difference between the postoperative angle and that measured at the final follow-up (*P* < 0.05) (Fig. 1).

Severity grading according to the American Spinal Injury Association grading system is presented in Table 3. All patients had suffered from an evident neurological deficit before surgery. Of the 69 patients, 62 (89.86%) showed complete neurological recovery. According to ASIA grading, five patients were classified as grade A, 15 as grade C and 49 patients as grade D preoperatively. This had improved postoperatively to five patients classified as grade C, 16 as grade D and 48 as grade E, and at the final follow-up four patients were grade C, three were grade D and 62 were grade E. There was a significant difference between the preoperative and postoperative grades (*P* < 0.05). The ODI scores decreased from 36 ± 9 on average prior to surgery to 20 ± 8 after surgery, with an average score at the final follow-up of 4 ± 3. The average preoperative and postoperative pVAS scores were 6.0 ± 1.5 (range 3–9) and 2.6 ± 0.9 (range 1–5), respectively. There was a significant difference between the preoperative and postoperative pVAS scores (*P* < 0.05). At the final follow-up visit, the average pVAS score was calculated to be 0.5 ± 0.6 (range from 0 to 2), and there was a significant difference between the postoperative and follow-up pVAS scores (*P* < 0.05). There were four patients presented with water-electrolyte imbalance after surgery. Two patients experienced transient worse neurological deficit (grade D preoperatively to grade C postoperatively) but complete neurological recovery at the last follow-up. Six patients presented with anti-tuberculosis drug-induced liver and kidney dysfunction. Other complications such as superficial or deep infection, cerebrospinal fluid leak, transpedicular screws loosening, slippage or fracture of graft, were not seen in patients of our study.

**Table 3:** The preoperative, postoperative and final follow up of neurological function was evaluated by ASIA grading

	Classification of neurological function by ASIA				
	A	B	C	D	E
Preoperative	5		15	49	
postoperative			5	16	48
Final follow up			4	3	62

## Discussion

Our research shows that single-stage posterior debridement, decompression and transpedicular screw fixation is an effective and safe approach for the treatment of patients presenting thoracolumbar junction (T12-L1) tuberculosis with neurological deficits. However, standard antituberculosis chemotherapy, strict bed-rest and supportive therapy remain the fundamental approaches for treating spinal tuberculosis. Surgical intervention is only recommended for thoracolumbar spinal tuberculosis patients with significant neurological dysfunction [26].

In the present study, we evaluated the outcome of one-stage posterior debridement, decompression and transpedicular screw fixation for the treatment of 69 patients with thoracolumbar junction (T12-L1) tuberculosis and associated neurological deficits. The average blood loss of patients during surgery was 354 ± 291 mL. This blood loss is lower than that reported by Rawall et al. [27], who reported an average blood loss of 419 mL. No perioperative complications appeared in the present study.

The ASIA scale scores recorded at the final follow-up visit were significantly better than the preoperative scores (*p* < 0.05). Significant improvement in neurological grading was also evident, with complete neurological recovery of 89.86% of patients. This result is comparable with that reported in the study by Rawall et al. [27] and better than that reported in the study by Zhang et al. [28]. The average pVAS scores of these cases decreased from 6.0 ± 1.5 preoperatively to 0.5 ± 0.6 at the final follow-up visit. The average preoperative and postoperative kyphotic Cobb angles recorded in the present study were 21 ± 9° and 9 ± 4°, respectively, which were significantly different (p < 0.05). No intraoperative complications appeared in any of our cases. Thus, our study showed a satisfactory outcome in regard to the neurological dysfunction suffered by patients with thoracolumbar junction tuberculosis who were treated by a single-stage posterior debridement, decompression and transpedicular screw fixation approach.

Tuberculosis mainly affects the anterior column of the spine [29], and the most common form involves only a single motion segment. The anterior approach is preferred for decompression and debridement in spinal tuberculosis [14] as it allows direct access to the lesion site, radical debridement, adequate decompression and reduced muscle trauma.

Many experts believe that patients treated by the anterior approach experience greater blood loss. Furthermore, the duration of the operation itself and the hospitalisation period is longer than that of the posterior approach. Many authors report increased surgical complications associated with the anterior approach, such as nerve and vascular injuries [30, 31]. The advantages of the posterior approach include reduced bleeding and shorter hospitalisation and operation durations, in addition to relief of spinal nerve compression, corrected spinal kyphosis, regained spinal stability and improved quality of life. Moreover, the posterior approach may be a better surgical method in patients with less involved spinal tuberculosis for the anterior column that is mainly affected by tuberculosis achieving spontaneous fusion [32, 33]. Additionally, posterior pedicle screw fixation may improve neurological recovery, as rigid stabilisation enhances neurological improvement in patients with traumatic spinal cord injury [34].

We are also aware of the potential risk of tuberculosis spreading to the healthy posterior regions, as posterior debridement can result in diffusion of infection and fistulas. The stability of the spine would theoretically be affected because the normal posterior column of the spine can be destroyed due to debridement and decompression in this procedure. Fortunately, these complications were not found in our study. Long-term follow-up is needed to closely monitor the development of these potential complications.

Our research had some limitations. Firstly, it was a retrospective study rather than a prospective study. Secondly, the sample size was small. In addition, all surgeries were performed by the respective surgical team of the six teaching hospitals, which may cause a certain degree of bias due to differences in proficiency and ability.

## Conclusions

Our study demonstrated that single-stage posterior debridement, decompression and transpedicular screw fixation can be an effective treatment method for most patients with thoracolumbar junction (T12-L1) tuberculosis and associated neurological deficits, with good neurologic recovery, avoidance of kyphosis progression, and few complications.

## Abbreviations

ASIA: American Spinal Injury Association
CRP: C-reactive protein
ESR: Erythrocyte sedimentation rate
HREZ: Isoniazid, rifampicin, ethambutol, and pyrazinamide
ODI: Oswestry disability index
pVAS: Pain visual analogue score
